# Copy Number Variation across European Populations

**DOI:** 10.1371/journal.pone.0023087

**Published:** 2011-08-04

**Authors:** Wanting Chen, Caroline Hayward, Alan F. Wright, Andrew A. Hicks, Veronique Vitart, Sara Knott, Sarah H. Wild, Peter P. Pramstaller, James F. Wilson, Igor Rudan, David J. Porteous

**Affiliations:** 1 Medical Genetics Section, Centre for Molecular Medicine, Institute of Genetics & Molecular Medicine, University of Edinburgh, Western General Hospital, Crewe Road South, Edinburgh, United Kingdom; 2 MRC Human Genetics Unit, Institute of Genetics and Molecular Medicine, Western General Hospital, Edinburgh, United Kingdom; 3 Institute of Genetic Medicine, European Academy Bozen/Bolzano (EURAC), Bolzano/Bozen, Italy - Affiliated Institute of the University of Lübeck, Lübeck, Germany; 4 Institute of Evolutionary Biology, University of Edinburgh, Ashworth Laboratories, King's Buildings, Edinburgh, United Kingdom; 5 Centre for Population Health Sciences, The University of Edinburgh Medical School, Edinburgh, United Kingdom; 6 Department of Neurology, General Central Hospital, Bolzano, Italy; 7 Department of Neurology, University of Lübeck, Lübeck, Germany; 8 Croatian Centre for Global Health, University of Split Medical School, Split, Croatia; Ohio State University Medical Center, United States of America

## Abstract

Genome analysis provides a powerful approach to test for evidence of genetic variation within and between geographical regions and local populations. Copy number variants which comprise insertions, deletions and duplications of genomic sequence provide one such convenient and informative source. Here, we investigate copy number variants from genome wide scans of single nucleotide polymorphisms in three European population isolates, the island of Vis in Croatia, the islands of Orkney in Scotland and the South Tyrol in Italy. We show that whereas the overall copy number variant frequencies are similar between populations, their distribution is highly specific to the population of origin, a finding which is supported by evidence for increased kinship correlation for specific copy number variants within populations.

## Introduction

Copy Number Variation (CNV) is defined here as DNA segments of 1 kb or longer in length and present at variable copy number in comparison with a reference genome [1]. CNVs are commonly found in the genomes of human and other species [2]–[5]. To date, 35% of the human genome demonstrates evidence of coverage by CNVs (Database of Genomic Variants, DGV, http://projects.tcag.ca/variation/). It is suggested that CNVs, in the form of deletions, insertions, duplications and complex multi-site variants, may contribute to human phenotypic variation, either directly by gene dosage and proportionate variation in gene expression [6], and/or indirectly through a) position effects on expression levels *per se* or developmental patterns of expression, or b) by affecting recombination rates and thus genome evolution [1]. Indeed, several studies have reported evidence for a direct contribution of CNVs to complex disease phenotypes in human populations, such as Schizophrenia and Autism [7]–[9], and in other species [10]–[16].

Copy number variation can be directly assayed by quantitation of hybridisation to specialist oligonucleotide [17], [18] or clone arrays [19] or by direct genome sequencing [20], [21], but also conveniently extracted from single nucleotide polymorphism (SNP) array data [22]–[24]. As well as being applied to the search for genetic contribution to disease phenotypes, several studies have provided global estimates of CNV frequency and distribution in HapMap samples [1], [6] and large population cohorts [22], [25]–[27], but relatively little attention has been given to potential variation within major population groups. Comparisons of CNV frequency and distribution between independent studies have also been hampered by discrepancies in study design, platform choice and analytical methods between studies.

Geographical population isolates are valuable resources for the dissection of complex genetic traits and disease outcomes [28]–[30]. Genetic isolates have reduced genetic heterogeneity, as measured by fewer net mutations and numbers of polymorphic SNPs compared with outbred populations [29]. Furthermore, by virtue of population bottlenecks, genetic drift and high kinship, each isolate will have a different evolutionary history and thus different genetic makeup. For example, isolate populations have been reported to show increased linkage disequilibrium and reduced haplotype diversity relative to outbred populations, consistent with reduced effective population size and increased genetic relatedness [31].

Here, we take the opportunity provided by the EUROSPAN project [32] which brings together several groups working on the genomic and phenotypic analysis of population isolates across Europe. Our objective was to make use of high density genome-wide genotyping data to describe and compare frequencies of each CNV and their distribution within and between these population isolates, and thus determine to what extent CNVs can be used as measures of relatedness and identifiers of population origin. Using Illumina whole genome data with more than 300,000 SNPs from each of three European population isolates, spanning from Northern to Southern Europe, we detected 4016 CNVs in 1964 individuals, which clustered into 743 copy number variable regions (CNVRs). The frequency and distribution of these CVNRs was compared and shown to differ significantly between the Orcadian, South Tyrolean and Dalmatian populations. Consistent with the inference that this indicated population-specific CNVR identity and origin, we also demonstrated that CNVR variation within each population can be used to measure genetic relatedness.

## Results

### Overview of copy number variation in Dalmatian, Orcadian and South Tyrolean populations

**Figure 1:**
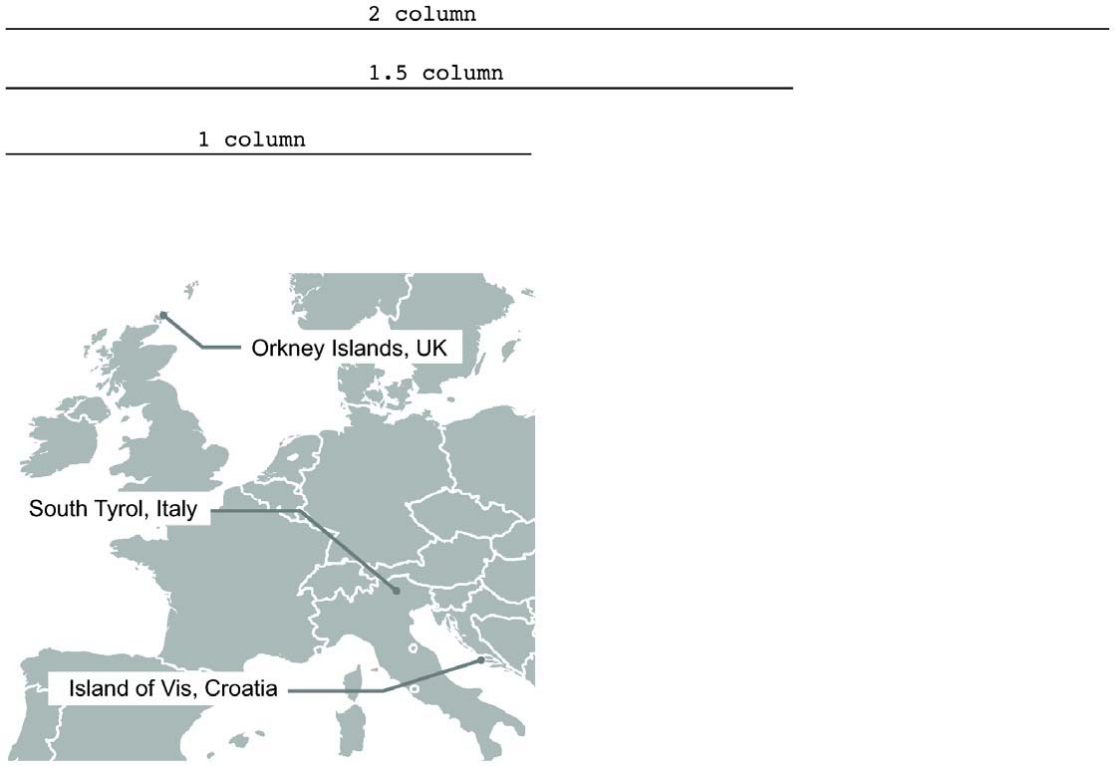
Geographic distribution of study samples.

The study samples were recruited from three populations across Europe, namely the Island of Vis, Croatia, Orkney Islands, Scotland and South Tyrol, Italy (Figure 1). 2789 individuals who passed quality control were included in the analysis. To generate more informative results [33], we utilized two algorithms, QuantiSNP [24] and cnvPartition to detect CNV events from SNP genotyping data. The combined analysis of CNV calling by QuantiSNP and cnvPartition software (see Methods) identified 4016 autosomal CNVs in 1964 individuals, out of the total 2789 samples, which makes 70.4% of them CNV carriers, with an average number of 2.05 detectable CNVs per carrier. 7.8% of the all autosomal SNPs were covered by CNVs. A correlation of SNP density and CNV length was observed, with higher SNP density in shorter CNVs and lower SNP density in longer CNVs (p<2.2*10).

**Figure 2:**
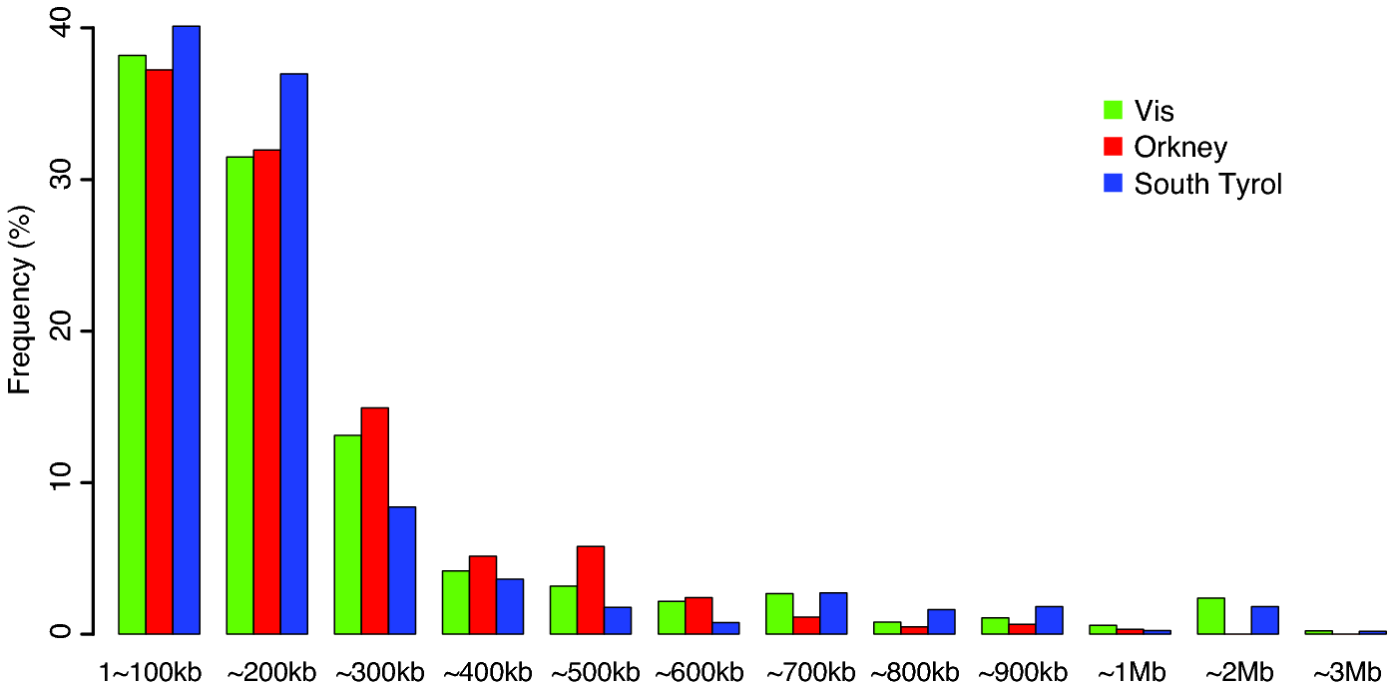
Distribution of CNV lengths in the three genetic isolate populations.

Fewer CNVs were detected on average in Orcadians (0.91 CNV per person) than in South Tyroleans (1.77 per person) or Vis islanders (1.43 per person). Equal numbers of amplification and deletion events were detected in each of the populations (Table 1). The overall length distributions of observed CNVs were also very similar between the three population isolates (Figure 2). Most CNVs were small in length (94.1% of the CNVs were between 1 kb to 300 kb, mean length was 205.1 kb, Table 1 and Figure 2).The lengths of amplifications (259 kb) were significantly greater (Mann-Whitney U test, P<2.2*10−16) than those of deletions (142.4 kb) (Table 1). 3778 out of 4016 CNVs (94.1%) overlapped with CNVs reported in the Database of Genomic Variants.

**Table 1:** Characteristics of Copy Number Variants (CNVs) in Dalmatian, Orcadian and South Tyrolean populations.

Population	Sample size	CNV carriers (percentage of carriers in population)	Number of CNVs	CNVs per person	Amplifications	Deletions	CNV mean length (kb)
**Vis**	965	702 (72.7%)	1384	1.43	803	581	216
**Orkney**	691	367 (53.1%)	630	0.91	324	306	192.6
**South Tyrol**	1133	895 (79.0%)	2002	1.77	1033	969	201.6
**Combined**	2789	1964(70.4%)	4016	1.44	2160	1856	205.1

The 4016 CNVs were clustered into 743 non redundant CNVRs (Table S1) which covered a total of 187.95 Mb (6.6%) of the 22 autosomes. 649 CNVRs (87.3%) overlap reported CNVs in DGV. Most of the CNVRs contained either only deletions or only amplifications, but 59 regions harbored both types of variants (Table 2). In these ‘gain-and-loss’ CNVRs, all of them contained at least one pair of CNVs whose boundaries were not equivalent from two individuals.

**Table 2:** Copy Number Variable Regions (CNVRs) in the three genetic isolate populations.

Population	Number of CNVRs	CNVRs overlapping reported regions	Number of deletion only CNVRs	Number of amplification only CNVRs	CNVRs of both deletion and amplification	CNVR mean length (kb)
Vis	365	332	184	164	17	304.5
Orkney	210	193	93	105	12	281.8
South Tyrol	380	334	156	207	17	256.9
Combined	743	649	323	361	59	253.0

### CNV frequency and CNV sharing among populations

Each CNVR was found in from 1 to 253 individuals, which made the overall frequency range of CNVRs to be from 0.00051 to 0.12882 (median = 0.00102). The CNVs identified were generally of low frequency. 337 CNVRs (45.4%) were detected in only one individual and 321 (43.2%) were shared by between 2 and 10 individuals. Only 37 CNVRs (5%) were present at a frequency >1% in all three population isolates.

**Figure 3:**
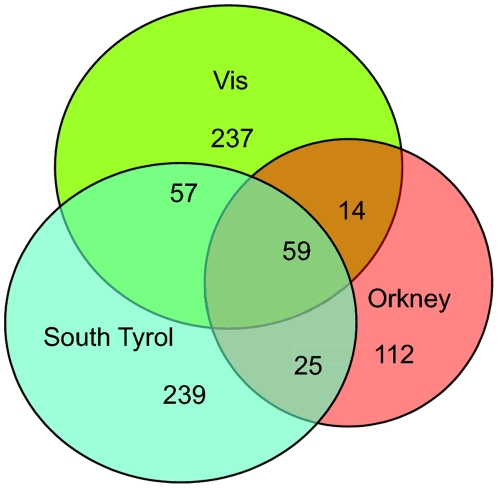
Venn diagram showing the number of CNVR shared between the three European genetic isolate populations.

**Figure 4:**
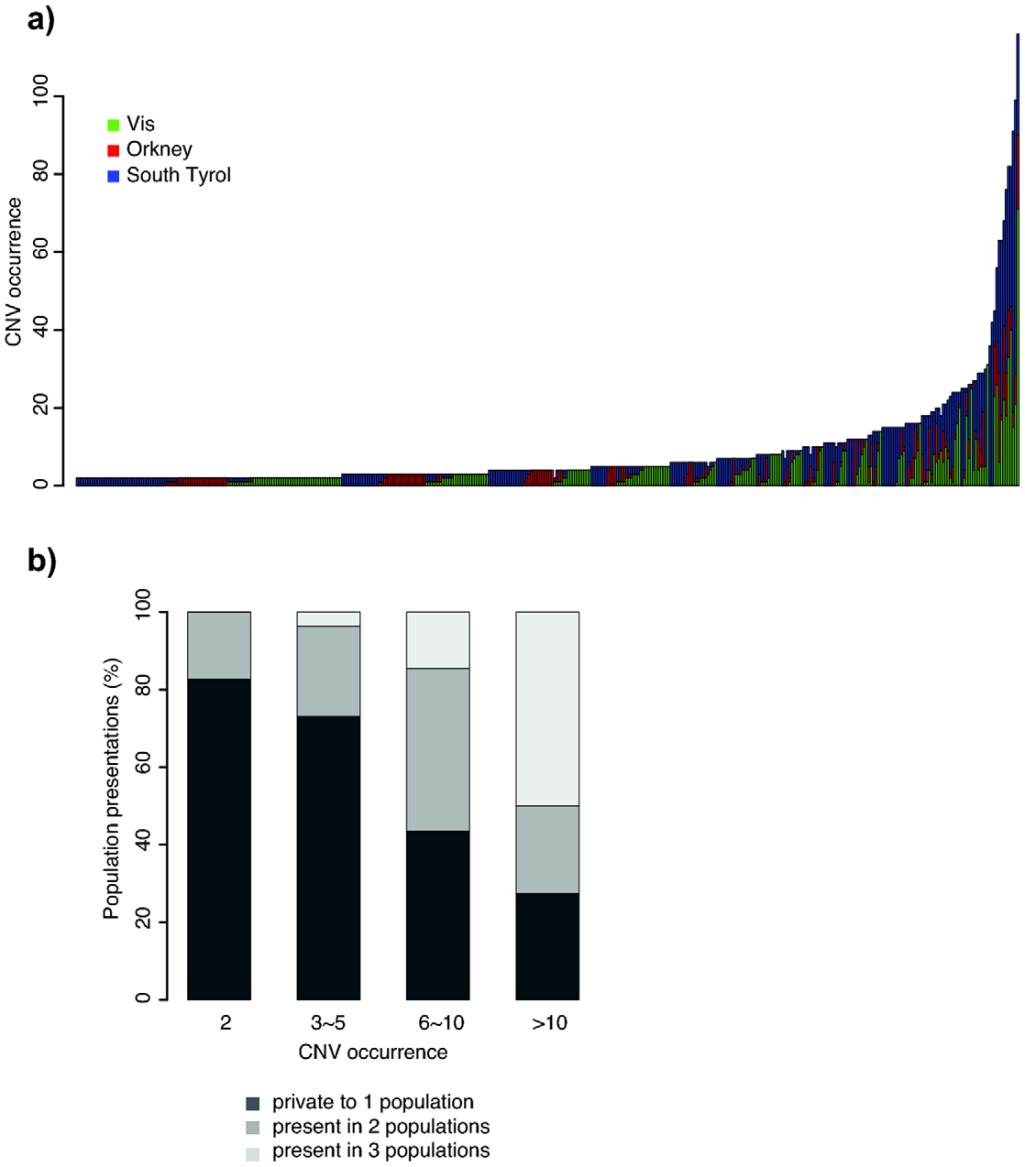
CNVR sharing in Dalmatian, Orcadian and South Tyrolean populations. (a) The population make up for each shared CNVR (shared by at least two individuals): each vertical bar represents for a CNVR, the height of each bar is the number of CNV carriers for each CNVR; colour blocks depict the proportions of CNV carriers from each of the three populations, green = Vis, red = Orkney, blue = South Tyrol. (b) Summary of population presentations for CNVRs of different frequencies: each bar represents a group of CNVRs of a certain frequency (from occurring twice to more than 10 times), different colours indicate the proportion of CNVRs private to only one population (in dark grey), CNVRs present in 2 populations (in grey) and CNVRs present in all 3 populations (in light grey).

Different patterns of CNV frequency were observed in different populations (Figure 3); 588 CNVRs (79.1%) were specific to just one of the three population isolates: 244 of them were detected only in Dalmatians, 112 only in Orcadians and 239 only in South Tyroleans; 96 CNVRs were shared by two of the three populations (57 between South Tyroleans and Dalmatians, 25 between South Tyroleans and Orcadians, and 14 between Dalmatians and Orcadians); and 59 were present in all three populations, non of which were novo. Less than half of these population-specific CNVRs (279 out of 588) were reported previously, according to DGV. Rare CNVs were found to be mostly restricted to a single population, while more frequent CNVs were often shared by two or three populations (Figure 4a). A gradual increase of population mixture was observed as the frequency of CNVRs increased: more common CNVRs were often shared in more than one population whereas lower frequency CNVRs were more likely to present in a single population (Figure 4b). The more frequent CNVRs in one population (population frequency>1%) were often observed to be also frequent in other populations. In South Tyrol, the frequencies of more common CNVs closely correlated with those of Dalmatian and Orcadian CNVs (Pearson's r = 0.73, P = 7.5*10−18 and r = 0.43, P = 0.005, respectively); the frequent Dalmatian CNVs also correlated with the frequent Orcadian and South Tyrolean CNVs (Pearson's r = 0.62, P = 0.001 and r = 0.65, P = 5.2*10−4, respectively), but there was no significant correlation between Orcadian and either Dalmatian or South Tyrolean CNVs of frequency>1% (Pearson's r = 0.38, P = 0.1347 and r = 0.22, P = 0.4046, respectively).

Of the 588 population specific CNVRs, more than half (337 CNVRs) contained only one CNV event. The mean length of CNVs in those population specific CNVRs was 250.3 kb, 205.5 kb and 195.6 kb in length, for Vis, Orkney and South Tyrol, respectively, which were on average longer than the ones for shared CNVRs (mean length 198.4 kb) (P = 0.04).

### Haplotype and SNP tagging for CNVs

To determine if the CNVs in our study sample were tagged by SNPs and to explore haplotype structure around CNVs, we carried out correlation analysis on the common CNVRs in Vis and Orkney samples (population frequency>1%): 2 of the 7 CNVRs in Vis, 1 of the 17 in Orkney and 15 of the 47 in South Tyrol were population specific, respectively. No tagging SNPs were found for any of these CNVRs with r^2^>0.8. 36 of these CNVRs overlapped CNVRs discovered in a large scale survey of tagging SNP for CNVs in UK samples [34]. Tagging SNPs were found in only 8 of these 36 regions. Haplotype block detection was performed for the 7 Vis and 17 Orkney CNVRs with SNPs 3 Mb upstream and downstream of each CNVR boundary. One CNVR (CNVR271, Chr6:67058287–67111682), could be placed in a haplotype block with 5 adjacent SNPs in all three populations. In addition, two population-specific CNVRs (CNVR367, Chr8:15987084–16065839 and CNVR386, Chr8:106005821–106293050) formed two haplotype blocks with nearby SNPs in the South Tyroleans.

### Genetic Clustering of individuals according to CNV genotypes

**Figure 5:**
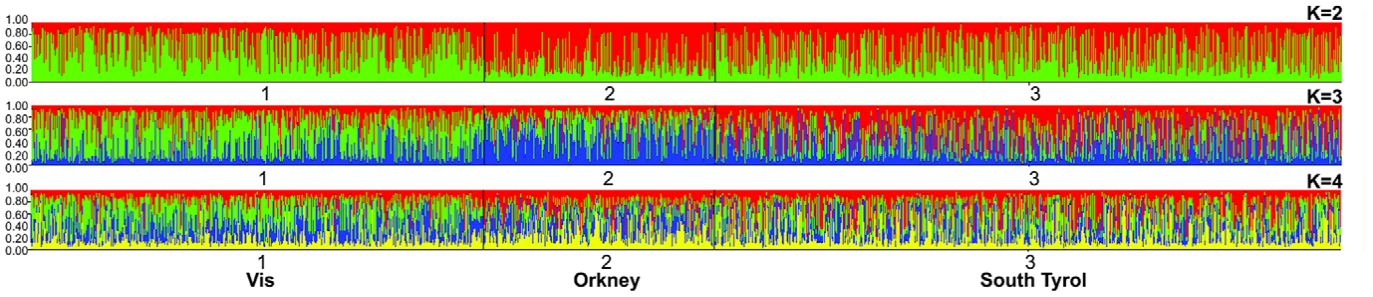
Genetic Clustering of individuals according to CNV genotypes. Cluster membership according to analyses of genotypes at 406 CNVR loci in 1893 individuals, for K = 2, 3 and 4. Each inferred cluster is represented by a different color. Cluster 1, Cluster 2 and Cluster 3 refers to Vis, Orkney and South Tyrol, respectively.

406 CNVR loci were observed multiple times in 1893 individuals (664 Dalmatians, 354 Orcadians and 875 South Tyroleans). Each of those loci were coded for these individuals as “CNV locus” or “non-CNV locus”, then software programme Structure [35] was used to determine how the individual clustered according to their possession of CNV. Graphical representation of membership in clusters for K = 2, 3 and 4 is shown in Figure 5. The distribution of the probability of the data between successive values of K showed a peak at K = 3, therefore it is inferred that the most likely number of genetic clusters for these individuals was three, with clusters roughly corresponding to the three geographical locations. 369 of 875 South Tyroleans (42%) were assigned to Cluster 1 (284 of them had membership coefficients≥0.5 for that cluster), 350 of 664 (52.7%) Dalmatians assigned to Cluster 2 (259 of them had membership coefficients≥0.5 for that cluster) and 179 of 354 (50.6%) Orcadians assigned to Cluster 3 (136 of them had membership coefficients≥0.5 for that cluster) (Table S2).

### Gene content

To test whether the detected CNVs were biased in any way towards genetic regions or were evenly distributed across the genome, the gene content of CNVs in the data set were investigated. 2211 CNVs in 441 CNVRs overlapped UCSC known genes. The mean number of genes covered by a CNV was 4.8, which was greater than the average gene content on autosomes (P = 0.00574). After introducing SNP density as a covariate into this regression model, the significance still remains (P = 0.00042). This result suggested a higher concentration of genes in CNVs. It was also found that the population specific CNVs overlapped more genes (on average 3.1) than that with the CNVs shared in more than one population (on average 2.3. p = 3.097*10^−5^). No elevated G+C content was detected (on average 40.41% in CNVRs) compared with the autosomal average G+C content (40.35%).

### Distribution along chromosomes

**Figure 6:**
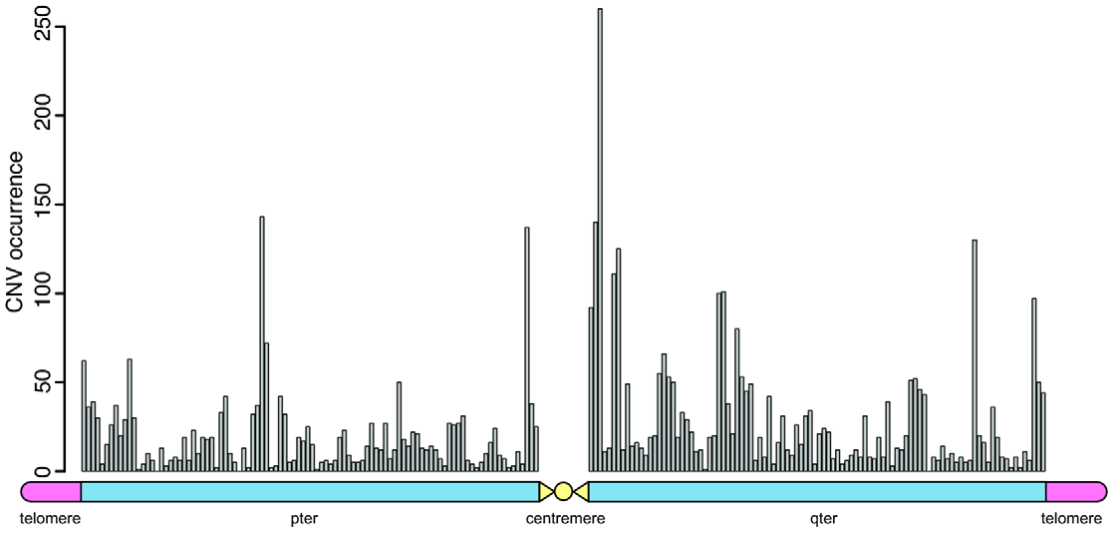
The schematic distribution of CNVs on all autosomes, in a physical map. The length of each chromosome arm is adjusted to be 100 Mb. Each bar comprises CNVs in a 1 Mbp bin on the chromosomes.

To test whether there was any bias in the overall chromosomal distribution of CNVs, we compared CNV density in pre-specified chromosomal regions (i.e. peri-telometric regions, defined as the 10 Mb region from the two most distal SNP on both chromosome ends and sub-centromeric regions, defined as the 10 Mb region from the two SNPs which were most close to centromere) to that in the rest of the chromosome. A trend was observed towards enrichment in peri-telomeric and/or sub-centromeric regions (Figure 6).

### Segmental duplications and CNVRs

Of the 743 CNVRs, 222 (98.1 Mb, 3.4% of all autosomes) overlap reported segmental duplications (SDs) or putative rearrangement hotspots: 102 CNVRs (41.3 Mb) overlap SDs but did not expand into the intervening regions between two SDs on the same chromosome; 153 CNVRs (68.5 Mb) were located in between two SDs of known rearrangement hotspots; the remaining 488 CNVRs (89.9 Mb) were not in SD regions or known rearrangement hotspot regions; of these 488, 409 (62.2 Mb) were population-specific.

Though no difference in G+C content was detected in CNVRs in general, a small increase of G+C content (41.79%) was found in CNVRs outside SDs, compared with that of CNVRs which overlap SDs (39.76%) (P = 1.78*10^−7^).

The proportion of CNVRs overlapping SDs was significantly lower for population-specific CNVRs (154 out of 588, 26.2%) than for shared CNVRs (68 of 155, 43.8%) (chi squared test, P<2.06*10^−16^).

### Kinship correlation of CNVs

We were interested to test whether carriers of shared CNVs showed more than average relatedness and developed a method to do so by incorporating a kinship coefficient, *k*, into the analysis (see Methods). The kinship coefficient is a parameter not dependent on population frequencies that measures the overall genetic similarity relative to some base population between a pair of individuals. For each CNVR with at least two carriers, the pair-wise kinship coefficients were calculated for all carrier pairs, then the value of those kinship coefficients were compared to the population mean of pair-wise kinship coefficients of all pairs of individuals in the corresponding population. It was observed that for most CNVRs (63.4% in Vis, 76.8% in Orkney and 83.4% in South Tyrol), CNV carriers had higher values of kinship coefficients compared to the population mean, indicating that carriers of shared CNVs are indeed more related to each other. (Table 3)

**Table 3:** Mean kinship coefficients of CNV carriers for CNVRs in three populations.

Population	Vis	Orkney	South Tyrol
**Mean ** ***k_pop_*** **(±s.d)**	0.000402±0.008027	0.001061±0.013336	0.001291±0.0137502
**Range of Mean ** ***k_n_***	0 to 0.3125	0 to 0.3125	0 to 0.3125
**Total CNVRs (of more than one carrier)**	172	112	205
**No. CNVRs with p_nadj_<0.05 (%)**	109(63.4%)	86(76.8%)	171(83.4%)

*k_pop_*, pair-wise kinship coefficients in one population. *k_n_*, pair-wise kinship coefficients of CNV carriers for the nth CNVR. p_nadj_ is the adjusted p value to describe significance of the differences of kinship coefficients among CNV carriers compared to the population mean pair-wise coefficients.

**Figure 7:**
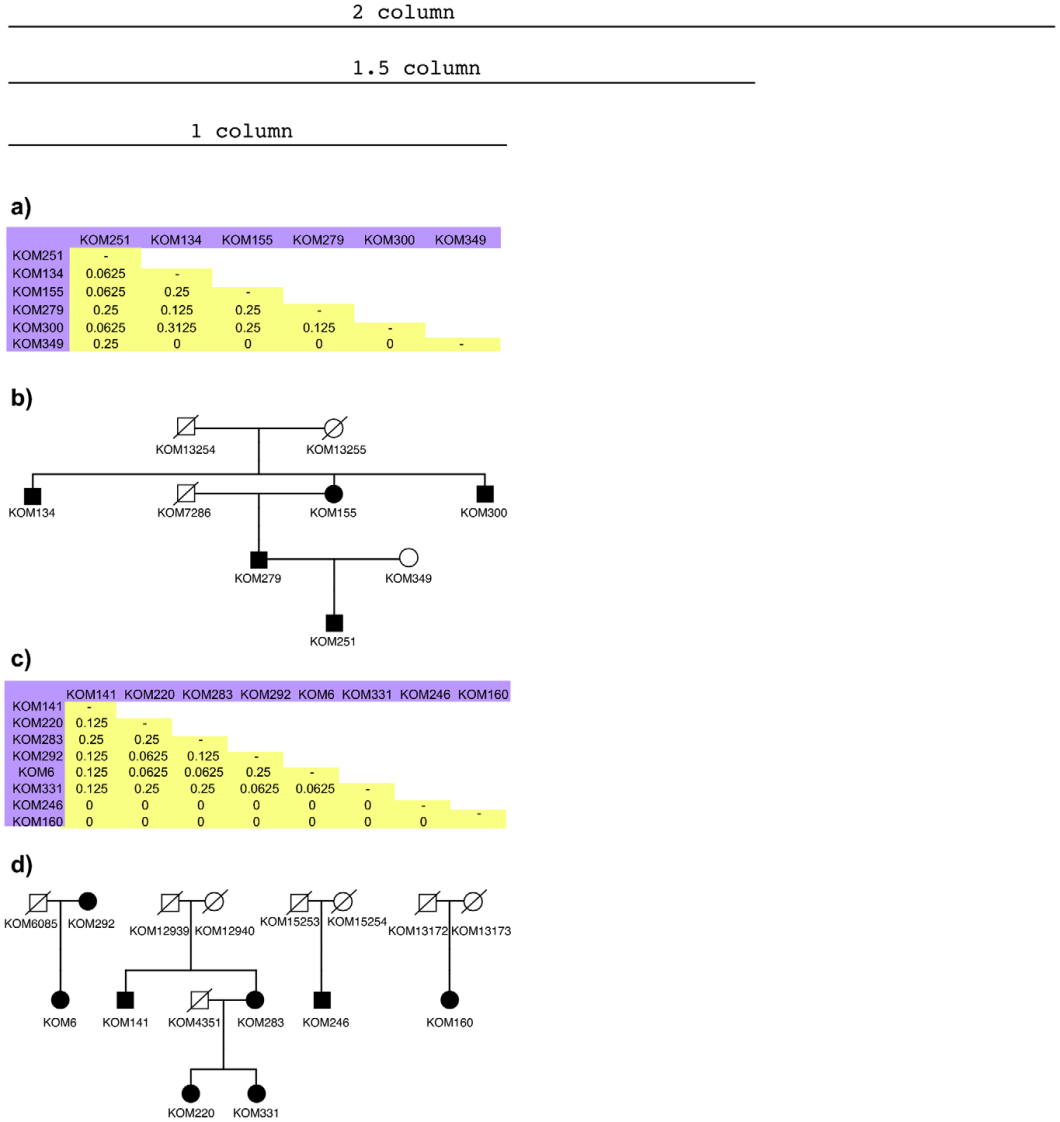
Two examples of segregation of CNVs in pedigrees: CNVR686 and CNVR54. (a) The kinship matrix of 6 carriers for CNVR686. They are all from the same population. The mean kinship coefficient of any pair of these 6 carriers is *k_691_* = 0.175, which is significantly higher than the population mean (adjusted p value<0.001) (b) The carriers for CNV686 placed in pedigree. Squares indicate male sex, circles indicate female sex. Filled squares or circles indicate CNV carriers. A cross through a square or a circle indicates the individual is either deceased or ungenotyped. (c) The kinship matrix of 8 carriers for CNVR54. They are all from the same population. The mean kinship coefficient of any pair of these 8 carriers is *k_55_* = 0.078, which is significantly higher than the population mean (adjusted p value<0.001) (d) The inheritance of CNV54. The key to the pedigree presentation is the same as for section (b).

Many CNVs with higher mean *k_n_* could be found to segregate in known families. Two examples were presented to illustrate the segregation of CNVs in pedigrees (Figure 7). CNVR686, an amplification on chromosome 19, was detected in 6 individuals who all turned out to had come from the same family (Figure 7 a) and b)). The inheritance pattern of this CNVR appeared to be autosomal dominant. CNVR54, a amplification on chromosome 2, was detected in 8 individuals. 4 of them were from the same known family, 2 of them were parent-offspring from another family while the other two were singletons (Figure 7 c) and d).

## Discussion

We profiled Copy Number Variation in three population isolates from UK, Italy and Croatia and representing a North-South, West-East geographical cline and components of the genetic diversity across Europe. This comparison of CNV characteristics was made possible by virtue of common choice of genotyping platform and copy number detection methods.

In common with previous reports from various populations and cohorts, we found that the great majority of individuals (70%) carried at least one CNV. CNVs were also widespread in the genome: 6.6% in length of all autosomal regions showed evidence of CNV in one or more samples. The proportion of SNPs covered by CNVs was 7.8%. The density of SNPs in CNVRs was 175.3 SNPs per Mb, while that in non-CNVRs was 117.1 SNPs per Mb (p<2.2*10^−16^). The lower density of SNPs in regions outside of detected CNVRs indicates that CNVs which reside in the SNP-sparse regions might not be captured on the commercial SNP genotyping platforms which lack coverage in certain chromosomal regions. The SNPs distribute more sparsely in longer CNV regions compared to those in shorter regions, therefore the boundaries determined for longer CNVs were less certain, which reflects the limitation of the HumanHap 300K arrays in terms of SNP coverage. A number of detected CNVRs were represented by both gains and losses. These ‘gain-and-loss’ CNVRs could reflect cases where the reference genome contains both CNV alleles, but individual genomes are homozygous for one or other allele. If true, then gains and losses within the same CNVRs should have equivalent boundaries. However, in all observed cases the gain-and-loss CNVRs in fact contained at least one pair of CNVs from two individuals whose boundaries are not equivalent. Although precise boundary determinations were subject to some technical uncertainty, it does appear that these gain-and-loss CNVRs most likely reflect recurrent CNV changes at the same locus, which are initiated and/or resolved at slightly different points.

Similar to other genetic polymorphisms such as microsatellites and SNPs, we show here that CNVs differ greatly among different populations. Indeed, the majority of CNVRs (588 out of 743 CNVRs) were restricted to one population and were often of very low frequency, their non-sharing across populations could be due to sampling variances or the fact that they were recent and/or possibly deleterious events. On the other hand, only the most frequently occurring CNVs, which were likely of more ancient origin, were shared between the three population isolates, consistent with a more ancient and neutral evolutionary histories, and also their geographic separation. The longer length and higher gene content of the population-specific CNVRs compared to those of the common CNVRs also supported the hypothesis that they may be more deleterious and therefore kept to low frequencies, or, those are more recent mutations that have had insufficient time to experience disruptive recombination events.

Whether SNPs can serve as a good proxy for CNVs has long been debated [1], [36]. Some studies suggested that deletion polymorphisms are generally in strong linkage disequilibrium and segregate on ancestral SNP haplotypes [34], [37], [38] while some others argue that although a number of CNVs are in strong linkage disequilibrium with nearby markers, accurate genotypes can only be captured for a small proportion of the tested CNVs [1]. We attempted to investigate LD between SNPs and CNVs, but due to the general low frequencies of the CNVRs in our populations, only a small number were available for testing. No tagging SNPs were found for 7 CNVRs in Vis, 17 CNVRs in Orkney and 47 CNVRs in South Tyrol. These CNVRs were also found to be poorly tagged by SNPs in the WTCCC samples [34]. Haplotype analysis revealed only three tagged CNVR, of which one CNVR (CNVR271, Chr6:67058287–67111682) was notable for being shared by all three populations. Analysis of an expanded set of CNVRs is warranted before firm conclusions on this issue can be drawn.

The CNV profiles in Vis and South Tyrol were more similar to each other compared to that of Orkney, in terms of number of shared CNVRs, correlation of CNV lengths and frequency. This may reflect their relative close geographical distances: Orkney is at 59 degrees north, whereas Vis and South Tyrol are both in Southern Europe.

Genetic clustering analysis formally demonstrated that CNVs can be used to classify the three population groups studied here and we can predict that the same will be true for other human populations, providing a potentially useful and applicable genomic tool for ancestry and evolutionary studies.

Consistent with other recent studies [39], [40], we found that CNVs tended to cluster in peri-telomeric/sub-centromeric regions, and commonly overlapped with segmental duplications and recombination hotspots, again consistent with the idea that they may serve well as ancestry markers.

As in many other studies [41]–[43], a higher gene content was discovered in CNVRs. It is argued that there is a high G+C content in gene rich regions [43], which are more frequently subject to copy number change. However, no elevated G+C content was detected in the observed CNVRs in this study. Although high gene content could be due to the bias of SNP choice in commercial genotyping arrays, after correcting for SNP density, the significance still remained. Some have argued that most of these genes are under negligible selective constraint; the CNVs influencing disease genes might have been eliminated by purifying selection. We also noted a significantly higher gene content within recent, population specific CNVRs. Further studies are warranted to test whether these are due to length of population specific CNVs being longer or they are under positive selection or can be linked (or elevated/diminished) to quantitative traits specifically in population isolates.

Finally, we show by the application of kinship coefficients that the majority of rare CNVs are passing through germ-lines rather than being *de novo* variants, and therefore are heritable and provide an index of relatedness. The inheritance of CNVs could be observed in actual pedigrees, which confirmed the increased relatedness between CNV carriers. The similar relationship between genetic variants and kinship was observed in a study of the same population in Vis, which found kinship inferred from pedigree information was consistent with segregation of SNPs in the population [44].

Illumina HumanHap300 SNP genotyping platforms were used to determine copy number variant events in our analysis. Despite the relatively lower SNP content of the 300K microarray compared with products such as Illumina Human 1 M and Affymetrix snp 6.0, the power of our method to detect CNVs from the 300K platform was adequate, and we were able to detect a large number of CNV events in the three isolated populations and draw conclusion of the differences between individuals from distinct communities in the context of CNV. However, it is argued that due to insufficient coverage of informative probes in certain chromosome regions (eg. gene sparse and segmental duplication regions) and the inability to discriminate higher number of copies (copy number>4) of a duplicated region for most CNV calling algorithms for SNP arrays, it is hard to accurately quantify the true extent of human copy number variation [23]. In light of whole genome sequencing project such as the 1000 Genome Project (http://www.1000genomes.org/), which provides a resource of whole genome sequences of multiple individuals [45], it is believed that we can benefit from high quality CNV detection directly from sequence data of samples, to better understand the diversity of CNVs within and between populations. In the meantime, mining the widely available SNP arrays coupled with family data of CNV calling represents a useful way of validating CNV calling and studying evolutionary history of CNVs.

## Materials and Methods

### Ethical approval and consent

Ethical approval was given for the patient recruitment in Vis, Orkney and South Tyrol by the relevant Research Ethics Committee of the Faculty of Medicine, University of Zagreb, Croatia, the Local Research Ethics Committee of NHS Orkney and the North of Scotland Research Ethics Committee in Aberdeen, and the Local Research Ethics Committee South Tyrol, respectively. In all three sites, volunteers gave written informed consent to all parts of the study with the research medical doctors or research nurse or research co-ordinator present to answer questions. They were made aware that they need not take part in all parts of the study and are free to withdraw at any time without consequences for them. In Orkney and Tyrol, volunteers chose whether to consent to their family doctor being contacted in the event of incidental findings coming to light. [32]

### Study sample

2789 individuals with data passing quality control (QC) from the island of Vis, Croatia (the Vis study [31], n = 965), the Orkney Isles, Scotland (The Orkney Complex Disease Study, ORCADES [27], n = 691) and South Tyrol, Italy (The Genetic Study of Three Population Micro-isolates in South Tyrol, MICROS [46], n = 1133) are included in the CNV analysis. The Orkney Complex Disease Study (ORCADES) is an ongoing family-based, cross-sectional study in the isolated Scottish archipelago of Orkney. Genetic diversity in this population is decreased compared to Mainland Scotland, consistent with the high levels of endogamy historically. Data for participants aged 18–100 years, from a subgroup of ten islands, were used for this analysis. The Dalmatian samples were recruited in the two villages of Vis and Komiza on Vis Island. The islands off the Dalmatian coast of Croatia have been the subject of extensive anthropological studies and those of more remote inhabitance, such as Vis Island, display an unusually high degree of isolation, which is supported by genetic structure study using short tandem repeat (STR) markers [31]. The Italian samples were recruited from the villages of Stelvio, Vallelunga and Martello in the South Tyrol, a mountainous region split between Italy and Austria. The geographical structure, historical and political events of this region resulted in the isolation of the population. Heterogeneity even between valleys of the same ethnic group was found, which was confirmed by phylogenetic analysis. These studies followed similar study procedures as part of the EU FP7 EUROSPAN study [32].

All three projects were approved by the relevant ethics committees. Data collection was carried out between 2003 and 2007 in the three locations. Informed consent and blood samples were received from all study participants.

### Genotyping

The Dalmatian samples were genotyped on the Illumina Infinium HumanHap 300 v1 platform while the Orcadian and South Tyrolean samples were genotyped on the Human Hap 300 v2 platform (Illumina, San Diego, CA, USA). Individuals with less than 90% call rate were removed. Sex checks and IBD sharing between first- and second-degree relative pairs were performed with the PLINK program (http://pngu.mgh.harvard.edu/purcell/plink/) [47], and individuals with discordant pedigree and genomic data or falling outside expected ranges were removed from the study. SNPs on the sex chromosomes were excluded. Finally 300,938, 309,200 and 308,396 SNPs remained in Dalmatian, Orcadian and South Tyrolean datasets, respectively.

### CNV calling

For each individual, the Log_2_R ratio and B allele frequency of each SNP were processed by QuantiSNP and cnvPartition software to generate CNV calls.

The two independent sets of CNV calls made for the same individual were then assessed. The output from QuantiSNP and cnvPartition both provide information for each CNV on the chromosome number and chromosomal coordinates of the start and end of each CNV (breakpoints). One sample processing >35 CNVs detected by cnvPartition was excluded from the further analysis. Genomic coordinates of each CNV detected in each person were mapped to hg18 sequence assembly using LiftOver (http://genome.ucsc.edu/cgi-bin/hgLiftOver).

SNP coverage in centromeric regions is very low, thus CNVs called in these regions are likely to be false positive. For this reason all the CNVs spanning centromeres were excluded from the analysis (according to the coordinates of centromeres on each chromosome). CNVs smaller than 1 kb or larger than 3 Mb were excluded.

QuantiSNP and cnvPartition outputs were combined to produce a list of sample wise CNVs. A confirmed CNV call was made if 1) the CNV was identified by both methods at the same locus and the overlap indicated by both methods exceeds 50% in length; 2) the type of a copy number change event (copy number loss or copy number gains) called by both methods was consistent and 3) overlap length was between 1000 bp and 3 Mbp. The boundaries of a CNV were taken as the beginning and end of the overlapped section.

To locate CNVs on chromosomes, individual-wise CNVs were merged into Copy Number Variable Regions (CNVRs). A CNVR is the maximum region shared among all individuals carrying a CNV at the same locus.

### Sensitivity and specificity of CNV detection

The method to assess sensitivity and specificity of CNV detection on the Illumina genotyping platform is described in a previous study [48]. False positive rate estimation was based on simulation of chromosome 1 data from a male sample. This sample was chosen because it passed all QC criteria recommended by the program authors (standard deviation of LRR<0.3 and standard deviation of BAF<0.15) and did not contain an unusually high number (>35) of putative CNVs detected (either by QuantiSNP or cnvPartition). The LRR and BAF for all SNPs on chromosome 1 were shuffled, then QuantiSNP was run on such randomized chromosome 1 data to make CNV detection. This process was repeated 1000 times. At LBF (a posterior measure of confidence in the call) filter set to 10, QuantiSNP detected 1 false positive CNV per 23,381,000 SNPs (1000 simulated chromosomes of 23381 SNPs).

False negatives were estimated by taking chromosome X segments from the same male individual, as these are hemizygous genotypes which could serve to represent deletions. LRR and BAF of 20 SNPs were selected from randomized chromosome X data and replaced LRR and BAF of 20 consecutive SNPs at a random location on each randomized chromosome 1. This artificially constructed chromosome was examined by QuantiSNP. This process was repeated 1000 times. 20 SNPs were chosen for the length of each pseudo deletion as the mean length of DNA segments spanning 20 SNPs (211 kb) on chromosome 1 was similar to the mean length of CNVs detected for all actual samples in our study. At an LBF cut-off of 10, the false negative rate was 2.6% (974 out of 1000 pseudo deletions were detected). The sensitivity to detect shorter CNVs was lower.

To further reduce the false positive rate and detect CNV calls with more certainty, a second algorithm, cnvPartition was applied to the same samples in our study. Only those CNVs detected by both algorithms could be included. 82% of QuantiSNP calls overlap those from cnvPartition.

### Haplotype and SNP tagging

9 and 22 CNVRs from Vis and Orkney, respectively, each with a population frequency of >1%, were analyzed with Plink (http://pngu.mgh.harvard.edu/~purcell/plink/) [47]. SNP genotyping data was exported from BeadStudio and merged with CNV genotypes of the same individuals. Tagging SNPs were investigated with a window size of 3 Mb spanning each CNVR. For each CNVR, the adjacent SNPs 1 Mb upstream and downstream to the genomic location of each CNVR were selected in haplotype analysis.

### Genetic clustering analysis

Genetic clusters of a selected set of CNVRs, in which each CNVR was shared by two or more individuals, were inferred by the software Structure [35], under assumptions of admixture, correlated allele frequencies and no prior population information. For each number of clusters (K) from 2 to 4, a burnin length of 10,000 iterations followed by 10,000 Markov Chain Monte Carlo iterations was used. The second order rate of change of logarithmic probability of data between subsequent K values was estimated to identify the optimal number of clusters in the data.

### Analysis of CNV kinship correlation

The kinship coefficient is a measure of overall genetic similarity relative to some base population in two diploid organisms.

For each population, P, with T individuals in total, suppose there are N CNVRs: CNVR_1_, CNVR_2_, …, CNVR_N_, each with M_1_, M_2_,…,M_N_ CNV carriers ({M}> = 2 and {M}<T). For the nth CNVR (1≤n≤N), CNVR_n_, there are Mn people carrying the same CNVR.

Extract a sub kinship matrix from the population kinship matrix with those carriers C_1_, C_2_, …, C_Mn_ for CNVRn:
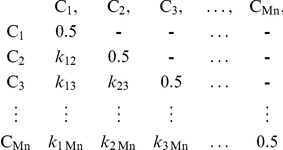
This is a Mn*Mn matrix, which is symmetrical around the diagonal line. Let *k_ij_* denote the pairwise kinship coefficient between individuals C_i_ and C_j_ (i = {1,2,3,…Mn}, j = {1,2,3,…Mn}). At the diagonal line of this matrix, *k_ij_*|i = j = 0.5, because when considering the probability of a random chosen allele to be IBD between two identical genomes, the same allele can be drawn twice.

In this sub-matrix for CNVRn, let *Kn* denote the non-redundant collection of all pair-wise kinship coefficients between any two individuals out of all Mn carriers.
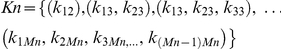
Let *Kpop* denote the non-redundant collection of all pair-wise kinship coefficients between any two individuals out of all T individuals in the population
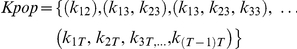
Therefore *Kn* has (Mn−1)! elements and *Kpop* has (T−1)! elements.

Then a t-test is performed to test the difference of means between *Kn* and *Kpop*. The probability, *p_n_* is calculated to indicate significance of this difference. A permutation procedure is taken to adjust *p_n_*: another Mn*Mn matrix is randomly drawn from population kinship matrix, with the pair-wise kinship coefficients
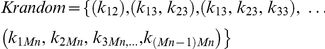
A p value, *p_perm_* is obtained from a t-test of comparing means of *Krandom* and *Kpop*. The same random process repeats 1000 times, result in 1000 *P_perm_* values. *p_n_* is then ranked among the permutated p values, the adjusted *p_n_*, *p_nadjust_* is the number of permutated p values which do not exceed *p_n_*, divided by the number of permutations.

### Statistical analysis

The reference CNV list was downloaded from DGV. The record of known genes and recombination rates in the human genome was downloaded from the UCSC genome browser. Intra- and inter-chromosomal segmental duplications (SDs) of >90 identity and >1 kb in length, which cover 150.8 Mbp of human genome (5.3%) [49], [50] were downloaded from Segmental Duplications Database (http://humanparalogy.gs.washington.edu/, build 36).

All calculations and alignments were performed with the R 2.10.1 software package. The test of difference in means was conducted using student's t-test for normalized data or the non-parametric Mann-Whitney U test, significant threshold set to 0.05.

## Supporting Information

Table S1 **List of Copy Number Variable Regions in three populations.**
(XLSX)

Table S2 **Membership coefficients for each cluster at k = 3.**
(XLSX)
